# Peripheral arterial blood pressure versus central crterial blood pressure monitoring in critically ill patients after Cardio-pulmonary Bypass

**DOI:** 10.12669/pjms.332.12220

**Published:** 2017

**Authors:** Rana Altaf Ahmad, Suhail Ahmad, Anjum Naveed, Mirza Ahmad Raza Baig

**Affiliations:** 1 Prof. Dr. Rana Altaf Ahmad, DA, FCPS, M. Sc. Pain Medicine, Executive Director, Professor of Anesthesia and Critical Care, CPE Institute of Cardiology, Multan, Pakistan; 2 Dr. Suhail Ahmad, DA, MCPS, FCPS, M. Sc. Pain Medicine, Associate Professor of Anesthesia and Critical Care, CPE Institute of Cardiology, Multan, Pakistan; 3 Dr. Anjum Naveed, FCPS, Assistant Professor of Pulmonology, CPE Institute of Cardiology, Multan, Pakistan; 4 Dr. Mirza Ahmad Raza Baig, B. Sc. Hons. CPT, Clinical Perfusionist, CPE Institute of Cardiology, Multan, Pakistan

**Keywords:** Cardiopulmonary bypass, Femoral artery, Invasive blood pressure, Radial artery

## Abstract

**Objective:**

To determine the accuracy of peripheral (radial) arterial access as compared to central (femoral) arterial access for measurement of invasive blood pressure (IBP) in critically ill patients after cardiopulmonary bypass.

**Methods:**

Sixty patients (60) who required high inotropic/vasopressor support on weaning from cardio-pulmonary bypass and weaned off in 2^nd^ attempt were included in this study. The duration of this study was from June 2015 to August 2016. Radial and femoral arterial access was achieved in all patients for simultaneous measurement of blood pressure. Arterial pressures were noted after 5, 15 and 30 minutes of weaning from cardiopulmonary bypass for both radial and femoral artery simultaneously.

**Results:**

Mean age of study patients was 56.48±11.17 years. 85% patients were male. There was significant difference in systolic blood pressure, diastolic blood pressure and mean arterial pressures between the radial artery and femoral artery cannulation. Mean arterial pressures were significantly high in femoral artery as compared to the radial artery. The mean arterial pressures after five minutes of weaning using central access were 76.28±10.21 mmHg versus 64.15±6.76 mmHg in peripheral arterial access (p-value <0.001). Similarly we also found significant difference in mean arterial pressures after 15 minutes of weaning from cardiopulmonary bypass 78.70±10.12 mmHg in central access versus 72.03±6.76 mmHg using peripheral arterial access (p-value <0.001). The difference in arterial pressures were less marked as compared to the previous differences after 30 minutes of weaning from cardiopulmonary bypass as compared to the earlier readings (p-value 0.001).

**Conclusion:**

Peripheral arterial pressures are unreliable in critically ill patients after cardiopulmonary bypass receiving high dose of inotropic drugs. Central arterial access should be used in these patients to get accurate estimates of patients’ blood pressure in early periods after cardiopulmonary bypass.

## INTRODUCTION

Monitoring of arterial blood pressure is very important for the evaluation of hemodynamic measurements as it gives a mandatory information about cardiovascular performance and hence tissue perfusion.^1^,^2^ The most common indication for invasive blood pressure (IBP) monitoring is continuous hemodynamic monitoring in critically ill patients, during high risk and major surgery, in patients with sepsis and in patients receiving vasoactive drugs or changes in blood volume or arterial tone and those with arrhythmias.^3^-^5^ IBP monitoring is a gold standard as it allows beat by beat measurement of patients’ blood pressure and vasoactive drugs response in these patients. Hence arterial blood monitoring should be as accurate as possible.

Radial artery cannulation is used in about 92.0% cases and femoral artery cannulation is the 2^nd^ most commonly used artery.^6^ However some clinicians prefer femoral artery access because of its lower rates of occlusion, thrombosis, and infectious complications.^7^ Many studies have compared the accuracy of peripheral blood pressure monitoring and central blood monitoring in cardiac surgery patients.^8^-^12^ These studies were conducted on hemodynamically stable patients and compared the accuracy of radial and femoral arterial access either in pre-cardiopulmonary bypass or during cardiopulmonary bypass phase but not in post-cardiopulmonary bypass period. Dorman et al. concluded that IBP monitoring using radial artery cannulation underestimates the central arterial pressure and femoral line allowed a significant reduction in infusion of vasoactive drugs in critically ill patients.^13^ The aim of the present study was to determine the accuracy of peripheral (radial) arterial access as compared to central (femoral) arterial access for measurement of IBP in critically sick patients after cardiopulmonary bypass.

## METHODS

This prospective study was conducted in Cardiac surgery unit of CPE Institute of Cardiology Multan. Sixty patients (60) who required high inotropic/vasopressor support on weaning from cardio-pulmonary bypass (CPB) and weaned off in 2^nd^ attempt from CPB were included in this study. The duration of this study was from June 2015 to August 2016. The following criteria was used for high inotropic support; epinephrine or norepinephrine >0.1 μg/kg/minute or dobutamine >10 μg/kg/minute. Patients requiring mild to moderate or no inotropic support on weaning from cardiopulmonary bypass and patients who required inotropic support before cardiopulmonary bypass were excluded. Ethical approval was taken before starting the research work.

In all patients radial artery was cannulated using a standard 20 G radial artery catheter (Vygon, ArterioSel, VYGON Ltd) for measurement of Invasive blood pressure during surgery. Radial artery of non-dominant hand was selected by 1^st^ performing the Allen’s test. On weaning from cardiopulmonary bypass if patient required high inotropic support then femoral artery of the opposite leg of radial artery before weaning the patient from cardiopulmonary bypass. Right femoral artery was cannulated using 16 G femoral arterial catheter (Vygon, ArterioSel, VYGON Ltd). Separate transducers were used for measurement of radial and femoral artery pressures simultaneously. The position of both transducers were set at the level of the right atrium and zeroed to atmospheric pressure before connecting with the arterial line. The whole system was flushed with normal saline to remove air and to check any leaks at connections. Rapid flush test was used to check damping co-efficient, kinking of arterial line, and mal-positioning of the catheters. The transducers were connected to the monitor for continuous monitoring of the arterial pressures.

Arterial blood pressure was noted for both radial and femoral artery immediately after 5, 15 and 30 minutes of weaning from cardiopulmonary bypass. Systolic arterial pressure, diastolic arterial pressure and mean arterial pressures were noted at these time intervals.

Data were analyzed using SPSS v23 software. Independent sample t-test statistics were used for comparison of arterial pressures taking p-value <0.05 as significant difference. Repeated measurement ANOVA was used to compare difference in mean arterial pressures in femoral and radial line for different time intervals.

## RESULTS

Sixty (60) patients who required high inotropic support on weaning from cardiopulmonary bypass were selected. Mean age of study patients was 56.48±11.17 years and 85% patients were male. There were 90% patients who underwent coronary artery bypass grafting and only 10% patients underwent valvular heart surgery. Mean cardiopulmonary bypass time was 124.6±22.7 minutes (Table-I).

**Table-I:** Baseline and Procedural Characteristics of Patients.

*Variable*	*Value*
Age (mean ± SD)	56.48 ±11.17
Male Gender (%)	51 (85)
Female Gender (%)	9 (15)

*Type of Operation (%)*	

CABG	54 (90)
MVR	4 (6.7)
AVR	1 (1.7)
DVR	1 (1.7)
CPB Time (minutes)	124.6±22.7

CABG:Coronary artery bypass grafting, MVR:Mitral valve replacement AVR:Aortic valve replacement, DVR:Double (aortic + mitral) valve replacement, CPB:cardiopulmonary bypass.

There was a significant difference in systolic blood pressure, diastolic blood pressure and mean arterial pressures between the radial artery and femoral artery cannulation after 5, 15 and 30 minutes of weaning from CPB. The mean arterial pressures after five minutes of weaning using femoral access were 76.28±10.21 mmHg versus 64.15±6.76 mmHg in radial arterial access (p-value <0.001). Similarly we also found significant difference in mean arterial pressures after 15 minutes of weaning from cardiopulmonary bypass 78.70±10.12 mmHg in femoral access versus 72.03±6.76 mmHg using peripheral arterial access (p-value <0.001). There was also a significant difference between mean arterial pressures in radial and femoral artery, but this difference was less marked as compared to the previous ones (p-value 0.001).

**Fig.1:**
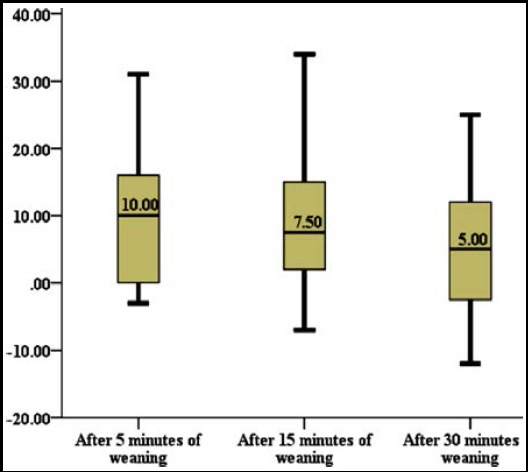
Difference between Mean Arterial Pressures of femoral and radial artery at different time intervals. The difference in mean arterial pressure were less and 30 minutes of weaning from CPB and highest at 5 minutes after weaning (P-value <0.001). This indicated significant reduction in difference in mean arterial pressures between the femoral and radial line with the passage of time. (Difference: MAP in femoral artery – MAP in radial artery).

**Fig.2:**
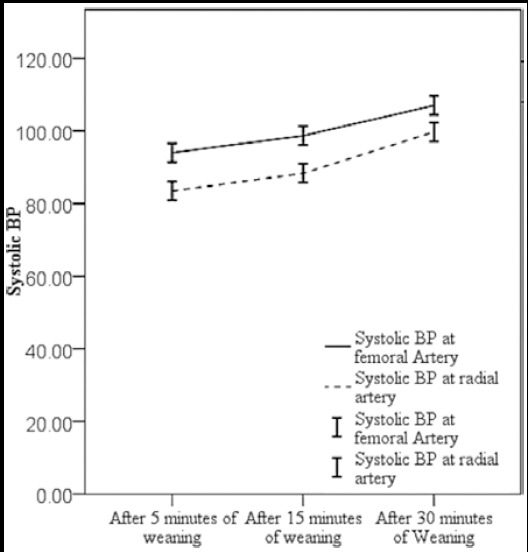
Graph Showing Systolic Blood Pressure at femoral and radial artery after 5, 15 and 30 minutes of weaning from Cardiopulmonary bypass. Difference in systolic BP was higher after 5 minutes of weaning and lesser after 30 minutes of weaning from cardiopulmonary bypass.

Similar trends were seen in systolic blood pressure of patients at femoral and radial artery, SBP were significantly high in femoral artery as compared to the radial artery (Fig.2). The difference between mean arterial pressures (MAP) of femoral and radial artery was also calculated and it was seen that the difference in mean arterial pressure were less after 30 minutes of weaning from CPB and highest at five minutes after weaning (p-value <0.001) (Fig.1).

## DISCUSSION

We concluded from the results of our study that femoral artery cannulation gives more accurate pressures recordings as compared to the radial artery cannulation in patients who become critically ill after cardiac surgery and require high inotropic/vasopressor support on weaning from cardiopulmonary bypass. On the contrary, O’Rouke et al. concluded that there is no difference in MAPs measured simultaneously in radial artery and the aorta in healthy volunteers.^14^

**Table-II:** Comparison of Arterial Blood Pressures between Radial and Femoral Artery.

	*Radial Artery Pressures*	*Femoral Artery Pressures*	*P-value*
Systolic BP after 5 minutes of weaning	83.46±8.29	94.01±10.49	<0.001
Diastolic BP after 5 minutes of weaning	53.28±7.82	63.55±9.49	<0.001
MAP after 5 minutes of weaning	64.15±6.76	76.28±10.21	<0.001
Systolic BP after 15 minutes of weaning	88.33±7.37	98.68±10.43	<0.001
Diastolic BP after 15 minutes of weaning	57.75±7.33	65.80±8.76	<0.001
MAP after 15 minutes of weaning	72.03±6.76	78.70±10.12	<0.001
Systolic BP after 30 minutes of weaning	99.73±10.58	107.01±10.60	<0.001
Diastolic BP after 30 minutes of weaning	67.18±9.12	71.05±8.22	0.02
MAP after 30 minutes of weaning	77.55±8.57	83.31±9.26	0.001

BP:Blood Pressure, MAP:Mean arterial pressures.

Chauhan and colleagues recommended that femoral arterial pressures are more accurate and reliable during cardiopulmonary bypass and monitoring of femoral arterial pressures results in lower doses of vasoactive drugs during CPB.^8^ Gravlee et al. showed that radial artery pressures are not reliable during initial phase of separation from cardiopulmonary bypass. They recommended that central arterial pressures e.g. aortic or femoral should be measured for 20 minutes after separation from cardiopulmonary bypass.^15^

Kim et al.^16^ conducted a study in septic shock patients receiving high dose of nor-epinephrine and concluded that radial artery pressures are unreliable in these patients and femoral artery pressures should be used in these patients. Routinely femoral arterial access is not used when other monitoring sites have failed. However, using femoral access the risk of ischemic complications and pseudo-aneurysmal formation is high afterwards.^17^,^18^ However, if we use femoral access for pressure monitoring only by inserting a smaller sized catheter there will be low risk of complications. The benefit of accurate monitoring of arterial pressures will outweigh the risk of complications.^8^,^19^

Fuda et al.^9^ concluded that smaller weight, smaller diameter of the radial artery, surgery in critically ill patients, complex surgeries, longer cross-clamp time and requirements of vasoactive drugs are major risk factors for central-to-radial arterial pressure gradient during cardiac surgery and femoral artery cannulation should be used in these patients for accurate measurement of invasive blood pressure.

Some other studies have concluded that arterial pressure gradients after cardiac surgery are associated with body temperature, bypass time and catecholamine levels.^11^,^20^,^21^ But other studies did not found the effect of these variables on arterial pressure gradients.^22^,^23^ Some studies have concluded that vasodilator therapy is beneficial in reducing arterial pressure gradients.^24^,^25^ Some studies have not found any significant effect of vasodilation or vasoconstriction of arterial pressure gradients.^23^,^26^ Even Urzua J found vasodilation as the main contributing factor of arterial pressure gradients after cardiac surgery.^27^

In our study, there was significant difference in mean arterial pressures between the peripheral and central arterial access. Similar trends were seen in systolic blood pressure and diastolic blood pressure in central versus peripheral access. There was a significant reduction in difference in mean arterial pressures between the femoral and radial artery with the passage of time and after 30 minutes of CPB this difference was only of 5 mmHg. So we concluded that peripheral arterial pressures are unreliable in critically sick patients after cardiopulmonary bypass receiving high dose of inotropic drugs. Central arterial access (via femoral artery of aorta) should be used in these patients to get accurate estimates of patients’ blood pressure in early period after cardiopulmonary bypass.

## CONCLUSION

Peripheral arterial pressures are unreliable in critically sick patients after cardiopulmonary bypass receiving high dose of inotropic drugs. Central arterial access should be used in these patients to get accurate estimates of patients’ blood pressure in early period after cardiopulmonary bypass.

### Authors’ contributions

**RAA:** Conceived, designed the research methodology and supervion of research work, and is accountable for all aspects of the work in ensuring that questions related to the accuracy or integrity of any part of the work are appropriately investigated and resolved. **SA:** Did data collection, helped in drafting and review the manuscript. **ANJ:** Drafted the manuscript and gave final approval of the manuscript to be published. **MARB:** Did data collection, analysis and interpretetion of results.
